# Anesthetic Management in a Post-COVID Hemolysis, Elevated Liver Enzymes, and Low Platelet Count (HELLP) Patient in Rural Central India: A Close Shave

**DOI:** 10.7759/cureus.24196

**Published:** 2022-04-16

**Authors:** Yatharth Bhardwaj, Vivek Chakole, Amol Singam, Sparsh Madaan

**Affiliations:** 1 Department of Anesthesia, Jawaharlal Nehru Medical College, Datta Meghe Institute of Medical Sciences (Deemed to be University), Wardha, IND; 2 Department of Obstetrics and Gynecology, Jawaharlal Nehru Medical College, Datta Meghe Institute of Medical Sciences (Deemed to be University), Wardha, IND

**Keywords:** maternal health, scoline, ketamine, general anesthesia, hellp syndrome

## Abstract

Hemolysis, elevated liver enzymes, and low platelet count (HELLP) syndrome is a dangerous complication of pregnancy amounting to significant mortality and morbidity. While conducting a cesarean delivery for such cases special care and consideration have to be taken in order to prevent maternal mortality. Thrombocytopenia is a significant abnormality that has to be weighed before induction in cases of HELLP syndrome with no clear cut-off value for the administration of neuraxial anesthesia. This case report aimed to explain the anesthetic management of a case with an indication for an emergency cesarean delivery.

## Introduction

Hemolysis, elevated liver enzymes, and low platelet count characterize HELLP syndrome, an advanced clinical stage of pre-eclampsia that results in significant maternal (24%) and newborn (up to 40%) mortality despite prompt delivery care [1]. The purpose of this study was to explain the anesthetic care of a case involving an emergency cesarean delivery. We report a case of a 33-year-old female patient, 67 kg with a gestational age of 36 weeks, after a possible diagnosis of post-coronavirus disease 2019 (COVID-19) HELLP syndrome. She arrived in an emergency operating room for a lower segment cesarean delivery. We chose intubation after rapid sequence induction with total intravenous induction with propofol, ketamine, and atracurium at doses of 2 mg/kg, 2 mg/kg, and 5 mg/kg, respectively, which was indicated for general anesthesia (all doses were calculated for patient's reference weight of 67 kg). Propofol and atracurium top-ups were used to keep the patient comfortable. The surgery went smoothly, and the baby was born and taken to the neonatal intensive care unit (NICU). The patient was extubated in the operation theatre at the commencement of the operation. The postoperative phase went without a hitch, with no noticeable alterations in primary vitals such as heart rate, respiratory rate, and blood pressure. Hence, when general anesthesia is recommended for a parturient amidst HELLP syndrome, rapid sequence induction with tracheal intubation, in addition to medicines to manage hemodynamic responses, aids to limit the procedure's hazards, as was the case in this case. This is an important aspect of patient care management, especially in the rural and remote areas of India when HELLP has emerged as an important post-COVID-19 sequela in the tiresome times of the ongoing pandemic.

## Case presentation

A 33-year-old female patient presented as G3P1L1A1 with a gestational age of 36 weeks, with a history of previous lower segment cesarian section and SARS-CoV-2 positive status 15 days back with thrombocytopenia and hyperbilirubinemia, and oligohydramnios with probable HELLP syndrome was referred to us for emergency cesarian delivery from the primary health care center. The patient had self-isolated herself at her residence for COVID-19 and had not done any blood investigations for the same. In the preoperative evaluation, pulse was 118 beats per minute with blood pressure measured to 100/60 mmHg and saturation 97% on room air. Preoperative laboratory investigations are shown in Table 1. The patient's prothrombin time and international normalized ratio (pt/inr) report were not available due to the sample being icteric and was not read by the lab machine.

**Table 1:** Laboratory investigations of the patient

Laboratory parameters	Measured values	Laboratory references
Hemoglobin	12.1 g/dl	Male: 13-15 g/dl; females: 11-13 g/dl
Total leucocyte count	11500/cubic millimeter	Normal: 4000-11000/cubic millimeter
Platelets count	45000/cubic millimeter	Normal: 1.5-4.5 lac/cubic millimeter
Alkaline phosphatase	537 U/l	Normal: 38-126 U/l
Aspartate aminotransferase (AST)	160 U/l	Males<17-59 U/l; females<14-36U/l; adult<15-46 U/l
Alanine aminotransferase (ALT)	150 U/l	Males<50 U/l; females<35 U/l
Total bilirubin	13.1 mg/dl	Normal: 0.2-1.3 mg/dl
Unconjugated bilirubin	1.7 mg/dl	Normal: 0.0-1.1 mg/dl
Conjugated bilirubin	11.4 mg/dl	Normal: 0.0-0.3 mg/dl
Total proteins	5.8 g/dl	Normal: 6.3-8.2 g/dl
Albumin	2.5 g/dl	Normal: 3.5-5.0 g/dl
Globulin	3.3 g/dl	Normal: 2.3-3.5 g/dl

**Figure 1:**
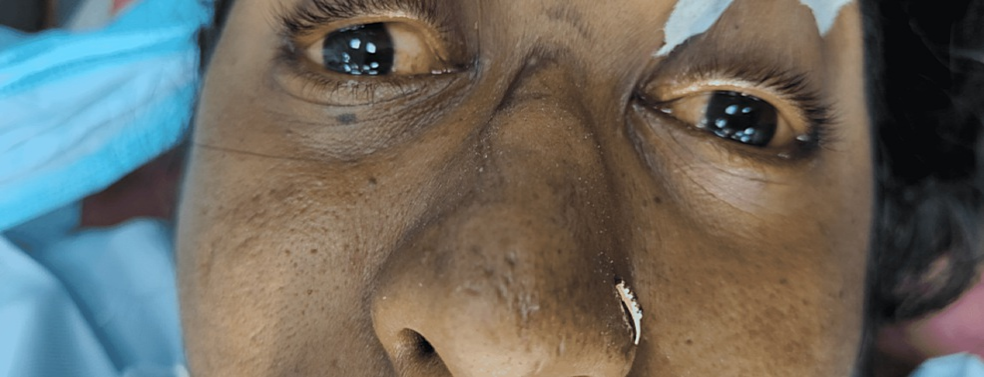
Icterus of the patient indicating the elevated bilirubin levels

**Figure 2:**
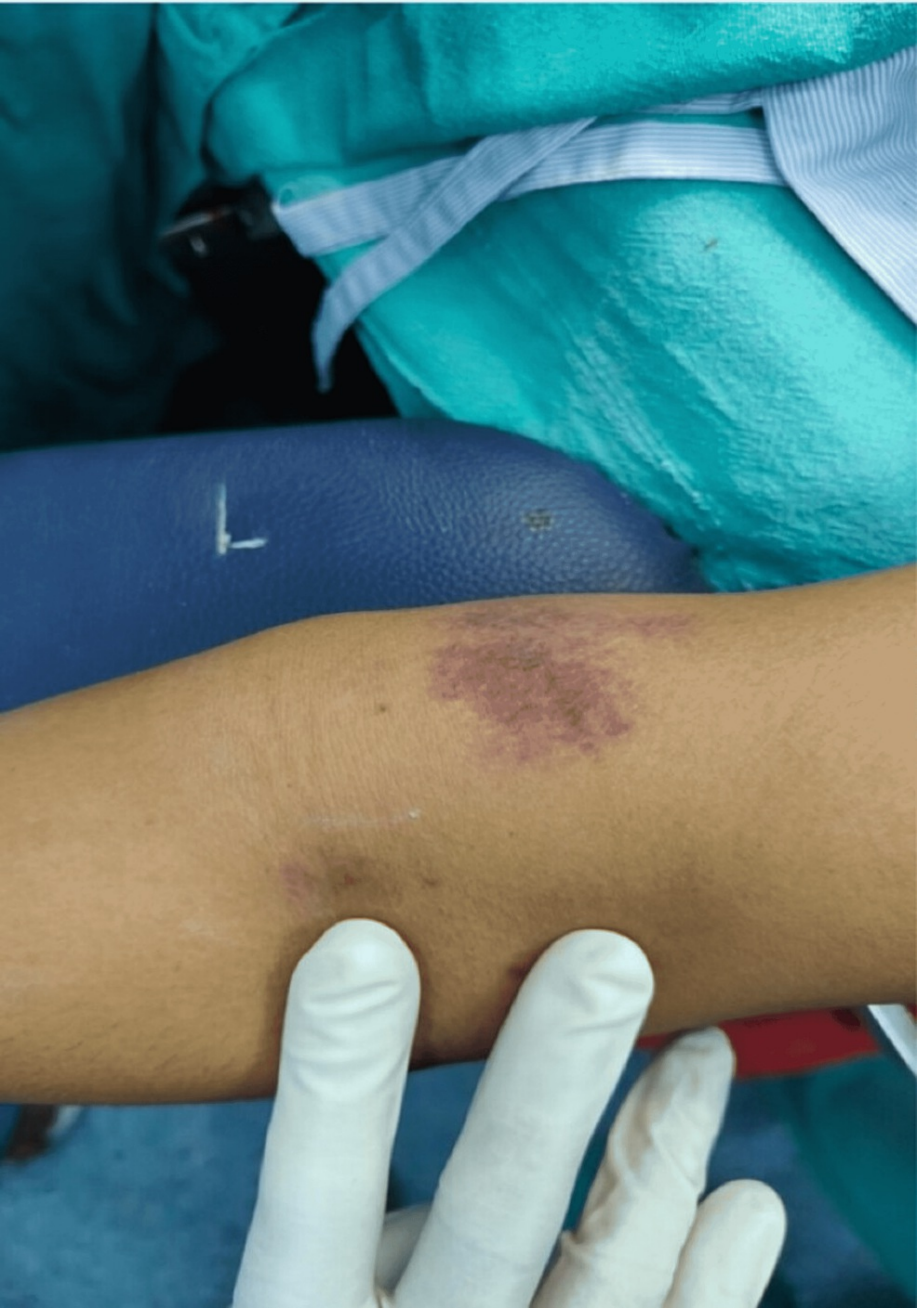
Petechiae over the patient's arm as a consequence of deranged platelet count

Airway examination revealed Mallampati classification of 2 and on auscultation notably, a reduced air entry in the right inframammary area was there compared to its left side. The patient on examination presented with a mild pallor and severe icterus (Figure 1), and petechiae were present with bilateral pedal edema (Figure 2). 

**Figure 3:**
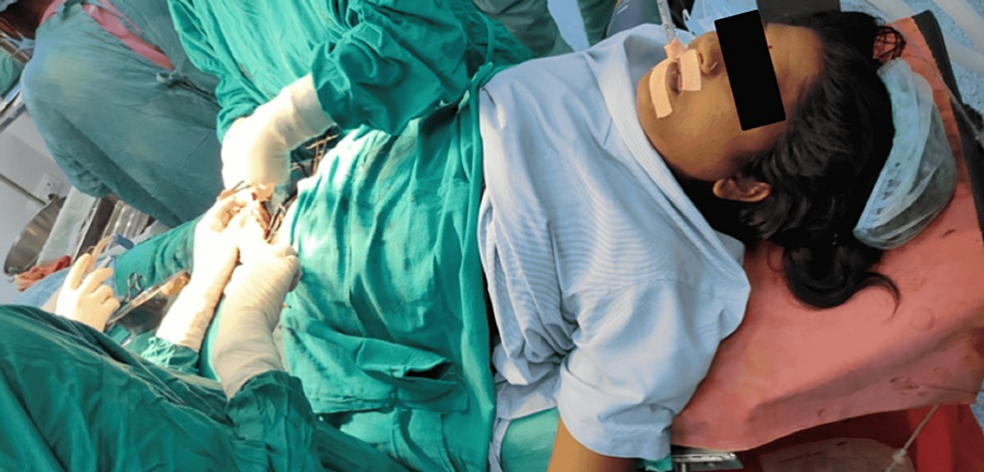
Intubated patient intraoperatively managed by rapid sequence intubation

After obtaining high-risk consent, the patient was taken for emergency cesarean delivery. General anesthesia technique was explained to the patient and the relatives. One unit of blood (packed red cell), four bags of fresh frozen plasma (FFP), and six bags of platelets were arranged under emergency without crossmatch for the surgery. Before induction, in both the upper limbs intravenous access was gained with a 16 G cannula and American Society of Anesthesiologist (ASA) standard monitors including monitors for pulse oximetry, non-invasive blood pressure monitoring, and ECG were connected. Supplemental oxygen via Hudson facemask is given. The patient and her relatives' consent were rechecked. The patient was induced with intravenous propofol 80 mg, intravenous ketamine 30 mg, and intravenous succinylcholine 100 mg given to facilitate the intubation. After watching for fasciculation, we intubated the patient with a 7.0 cuffed endotracheal tube. The patient was having Cormack-Lehane grading 1 as seen by laryngoscope blade number 3, and the tube was secured at the marking of 20 cm (Figure 3).

The patient was immediately connected with the anesthesia machine circuit with the volume control ventilation mode where tidal volume was set as 350 ml/min, with respiratory rate of 14/min. The surgeon also took all the precautions to not to delay the delivery and hastily proceeded to deliver the baby; as soon as the baby was delivered, the patient was given intravenous midazolam 1 mg, and inj fentanyl 50 µg, with inj Pitocin 20 international units which were put in the maintenance intravenous fluid, i.e., in 0.9% NaCl. All of the transfusion bags of platelets were thoroughly checked and blood groups with bag numbers were matched with the given file record, and one after the other six platelets were transfused as the patient blood loss was approximately 1000 ml, and the patient in total was given 1500 ml of intravenous 0.9% NaCl with ringer lactate used alternatively. The patient for the entire surgery was maintained on atracurium top-ups (5 mg/cc); as soon as the closure of the skin started, we prepared for the patient to be extubated, and after proper dressing of the incision site by the surgeon, the patient's mechanical ventilator support was called off and the patient was put off from the ventilator. The patient went on to take spontaneous breaths with generating adequate tidal volume, intravenous glycopyrrolate + intravenous neostigmine dose of 0.5 mg/kg was given slowly, as the extubation criteria were met, the patient was extubated smoothly and vital signs were stable postextubation.

## Discussion

HELLP syndrome is caused by aberrant placental development, which leads to the production of substances that enhance endothelial damage via platelet and/or vasoconstriction activation. Injury to the endothelium of hepatic arteries, followed by activation of platelets, aggregation, and consumption, culminating in hepatocyte which leads to ischemia and death, is the core idea to explain the characteristic laboratory results of HELLP syndrome. The judgment to employ anesthesia for preeclampsia patients is based on a comprehensive assessment of the advantages and hazards that anesthesia can provide to both mothers and newborns [2]. With neuraxial anesthetic procedures where coagulopathy along with thrombocytopenia, increases the risk of epidural hematoma [3]. Granted that there is no statistical data in the literature on the effects of neuraxial blockade in patients with HELLP syndrome and platelet counts below the specified value, guidelines recommend a platelet count of 100,000 mm to limit the risk [4].

When general anesthesia is applied, a combination of rapid sequence intubation, potentially difficult airway examination, and drugs capable to manage the hemodynamic response can help to limit the procedure's complications [5]. Alfentanil, fentanyl, remifentanil, lidocaine, and esmolol are the important drugs among them. In high-risk patients, remifentanil is frequently utilized to provide short-term analgesia while maintaining cardiovascular stability. Fentanil was administered for induction and maintenance of anesthesia in the situation mentioned here. In a recent survey, three case reports of patients with said syndrome and severe thrombocytopenia who received general anesthesia with sevoflurane instead of the epidural spinal block during cesarean delivery had excellent results. None of the complications documented in the literature (renal failure, pulmonary edema, cerebral hemorrhage, and hepatic rupture) were observed in the three postoperative days in the reported cases [6].

We have reported a case of HELLP syndrome which was referred from the primary health care. The patient had contracted COVID-19 about 15 days back which makes the probability of post-COVID-19 HELLP syndrome a likely differential. COVID-19 leads to thrombocytopenia due to damage to the endothelium, activation and aggregation of the platelets, bone marrow suppression, and impairment of the activity of the megakaryocytes [7]. This can have a presentation similar to HELLP syndrome. There have been prior reports of HELLP syndrome as long COVID sequelae due to prolonged inflammation with a fatal outcome indicating HELLP to be a possible post-COVID sequela [8]. As the patient belonged to a rural and remote area, she had not got any blood investigations done and was not on any follow-up for the same to monitor her inflammatory markers, platelet count, and liver enzymes. This highlights the importance of generating awareness and increasing vigilance in remote areas of India regarding post-COVID-19 sequelae.

As patients are regularly referred in rural areas due to HELLP syndrome with a lack of proper protocol for anesthesia management, it is important for such cases to be reported in order to aid the anesthetists and obstetricians working at the grass route levels of India. This shall not only help the clinicians in managing emergencies but also the community in reducing maternal mortality.

Limitation

A limitation in our case is that ideally an arterial line should be placed in such patients in order to foresee and manage the complications which may arise. However, due to the low socioeconomic status of our patient, this could not be done.

## Conclusions

Current research on the anesthetic management of people suffering from HELLP syndrome who need to have a cesarean delivery isn't clear on the optimum technique. This is essential in the current times when COVID-19 and HELLP overlap and associations have begun to present in the emergency department puzzling the clinicians globally. Given the potential for problems, it comes into view that general anesthesia with airway management following an induction in the rapid sequence is a good option. The pharmacological arsenal at hand should be carefully used, with special heed paid to drugs that promote surgical stability. Nevertheless, more research is needed to determine the optimum approach to utilize in patients with HELLP syndrome based on evidence.
